# Causal Effect of Blood Pressure on Bone Mineral Density and Fracture: A Mendelian Randomization Study

**DOI:** 10.3389/fendo.2021.716681

**Published:** 2021-08-04

**Authors:** Bin He, Lifeng Yin, Muzi Zhang, Qiong Lyu, Zhengxue Quan, Yunsheng Ou

**Affiliations:** ^1^Department of Orthopedics, The First Affiliated Hospital of Chongqing Medical University, Chongqing, China; ^2^Department of General Practice, The First Affiliated Hospital of Chongqing Medical University, Chongqing, China

**Keywords:** blood pressure, bone mineral density, fall, fracture, Mendelian randomization study

## Abstract

**Background:**

Hypertension may have some association with osteoporosis. This Mendelian randomization (MR) study aimed to explore the causal effect of blood pressure (BP) on bone mineral density (BMD), fall, and fracture.

**Methods:**

We used the genome-wide association study (GWAS) summary data among 330,956 European-descent individuals to identify 107 single-nucleotide polymorphisms (SNPs) as the instrumental variables of BP. MR analyses of these instruments were performed on 53,236 European individuals for the association with forearm BMD (FA-BMD), femoral neck BMD (FN-BMD), and lumbar spine BMD (LS-BMD); 451,179 European individuals for fall susceptibility; and up to 1.2 million individuals from European descent for fracture. Conventional inverse variance weighted (IVW) method was adopted to obtain the causal estimates of BP on different outcomes, while weighted median, MR-egger, and MR pleiotropy residual sum and outlier (MR-PRESSO) test were used for sensitivity analyses.

**Results:**

Genetically high pulse pressure (PP) could significantly improve FA-BMD (beta-estimate: 0.038, 95% confidence interval [CI]: 0.013 to 0.063, SE:0.013, P-value=0.003<Bonferroni correction P) in the IVW analysis, indicating that 1-SD increase in PP was associated with the improvement in FA-BMD levels by 0.038 g/cm^2^ (95% CI: 0.013 to 0.063). This positive finding was also confirmed by weighted-median analysis (beta-estimate: 0.034, 95% CI: 0.000 to 0.067, SE:0.017, P-value=0.046) and MR-Egger analysis (beta-estimate: 0.117, 95% CI: 0.026 to 0.208, SE:0.046, P-value=0.011). However, there was no remarkable MR association between BP and other outcomes (i.e., FN-BMD, LS-BMD, fall, and fracture).

**Conclusions:**

Our findings reveal a potentially causal relationship between high PP and improved FA-BMD, which may provide new sights for the treatment of osteoporosis.

## Introduction

Osteoporosis is one common systemic skeletal disease characterized by decreased bone mineral density (BMD) and increased risk of fracture (1–3). Its prevention and treatment are still a big challenge and growing public health problem in the world (4–6). Genome-wide association study (GWAS) has demonstrated that BMD is a highly polygenic trait, and some genetic determinants of fracture act through low BMD (7–9).

Hypertension is one common and strongly heritable disease, with high mortality and morbidity (10–12). It may also increase the risk of stroke and coronary artery disease (12, 13). Several observational studies reported the association between hypertension and BMD, but their results were conflicting (14–16). Mendelian randomization (MR) has become an effective and powerful approach to establish the causal relationships between exposure phenotype and outcome phenotype (17, 18). The use of MR study can overcome the limitations of confounding factors and reverse causation bias that commonly occur in observational studies (19, 20).

In this two-sample MR analysis, we used single-nucleotide polymorphisms (SNPs) strongly associated with systolic blood pressure (SBP), diastolic blood pressure (DBP), and pulse pressure (PP) as instrumental variables. To our knowledge, this is the first two-sample MR study to explore the causal effect of blood pressure (BP) on forearm BMD (FA-BMD), femoral neck BMD (FN-BMD), lumbar spine BMD (LS-BMD), fall, and fracture.

## Materials and Methods

This MR study was conducted based on the Strengthening the Reporting of Observational Studies in Epidemiology (STROBE) guideline (**Supplementary Table 1**).

### GWAS Summary Statistics of Blood Pressure

In the meta-analysis of genome-wide association study (GWAS) among 330,956 European-descent individuals, a threshold of P < 5*10^−8^ was used to denote genome-wide significance of blood pressure. The replication resources included a large blood pressure (BP) meta-analysis consortium (ICBP cohorts) and further cohorts with 1,000 Genomes data for GWAS (21). Blood pressure was defined as the mean arterial pressure with the lowest mean velocity index and estimated by the Omron device. Initially, 107 independent single-nucleotide polymorphisms (SNPs) were identified to have robust association with blood pressure at the GWAS threshold of statistical significance (P<5×10^−8^), including 24 SNPs for systolic blood pressure (SBP), 41 SNPs for diastolic blood pressure (DBP), and 42 SNPs for pulse pressure (PP) (**Supplementary Table 2**). These SNPs were all adjusted by the Principal Component Analysis in order to address the population stratification.

We excluded SNPs in strong linkage disequilibrium (LD) because they produce some bias. The clumping process (R^2^<0.001, window size =10,000 kb) was conducted with the European samples from the 1,000 genomes project, and we estimated LD between SNPs. Among the pairs of SNPs with r^2^≥0.001, the SNP with a larger association P value would be excluded. The SNPs that were absent from the LD reference panel were also removed. Thus, three SNPs about SBP, four SNPs about DBP, and five SNPs about PP were excluded due to LD. Finally, 21 SNPs for SBP, 37 SNPs for DBP, and 37 SNPs for PP were used as the instrumental variables (**Supplementary Table 3**). If SNPs were unavailable in the outcome dataset, the proxy SNPs in linkage disequilibrium (LD, r^2^>0.9) were used as the instrumental variables.

### Outcome Data Sources

A large meta-analysis was conducted to explore the genetic variants associated with FA-BMD, FN-BMD, and LS-BMD among 53,236 individuals of European ancestry (22). BMD was measured at the trabecular structure of forearm (distal 1/3 of radius), femoral neck, and lumbar spine (L1-4). BMD was measured by dual X-ray absorptiometry, and low BMD was defined as the Z-score <−1.0. Each SNP was tested after adjusting for sex, age, age^2^, and weight (22). A genome-wide association analysis involved 89,076 fall cases and 362,103 controls from the UK Biobank Study of European ancestry, and revealed the genetic determinants of fall susceptibility. Fall cases were defined as participants who gave positive answer to the following question: “In the last year have you had any falls?” (23). Genetic determinants of fracture risk were revealed in one large GWAS meta-analysis in up to 1.2 million individuals combining the UK Biobank and 23andMe cohorts. Fractures were defined as a break in the continuity of the bone at any site except the fractures of skull, face, hands and feet, pathological fractures due to malignancy, atypical femoral fractures, periprosthetic and healed fracture codes. The diagnosis of fracture should be within the past five years. (1).

### Statistical Analysis

We evaluated the causal effect of BP (SBP, DBP, and PP) on BMD (FA-BMD, FN-BMD, LS-BMD), fall, and fracture. MR estimates for instrumental variables were meta-analyzed by computing an inverse variance weighted (IVW) analysis for the primary analysis (24). We used Cochran’s Q analysis to assess the heterogeneity (25), where high heterogeneity indicated the presence of invalid genetic variants (26). For the sensitivity analysis, weighted-median analysis was conducted, which provided a valid estimate if at least 50% of weight originated from non-pleiotropic SNPs (27).

To assess the potential violation of these assumptions, MR-Egger analysis was used to assess the directional pleiotropy based on the intercept (28). The presence of pleiotropy was also assessed by the MR pleiotropy residual sum and outlier test (MR-PRESSO), during which outlying SNPs were excluded and the effect estimates were reassessed (29). The ethical approval and informed consent for each study included in the study can be found in the original publications. The differences with P<0.05 were considered statistically significant. In multiple testing, an adjusted P value after Bonferroni correction (P<0.05/4 = 0.0125) was considered statistically significant. All of the analyses were conducted in R V.4.0.4 by using the R packages of “MendelianRandomization” (30), “TwoSampleMR” (31), and “MR-PRESSO” (29).

### Role of the Funding Source

The funders of this study had an important role in study design, data collection, data analysis, data interpretation, and writing of the report. All authors had full access to all data in the study and had final responsibility for the decision to submit for publication.

## Results

### Causal Effect of Blood Pressure on BMD

**Figure 1:**
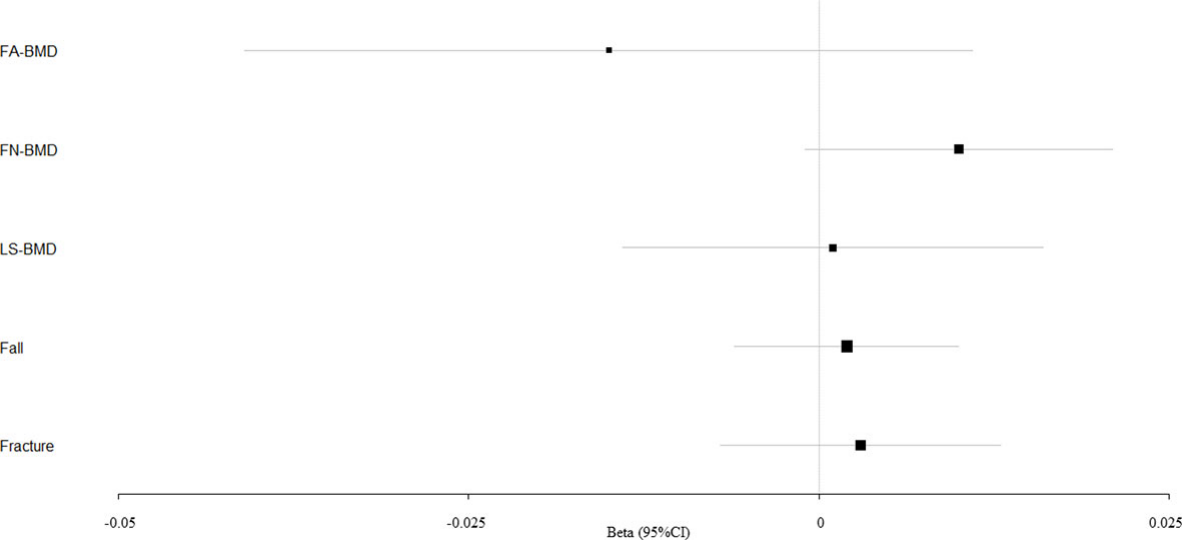
Mendelian randomization estimates for the associations between SBP and outcomes. FA-BMD, forearm BMD; FN-BMD, femoral neck BMD; LS-BMD, lumbar spine BMD; CI, confidence interval.

**Figure 2:**
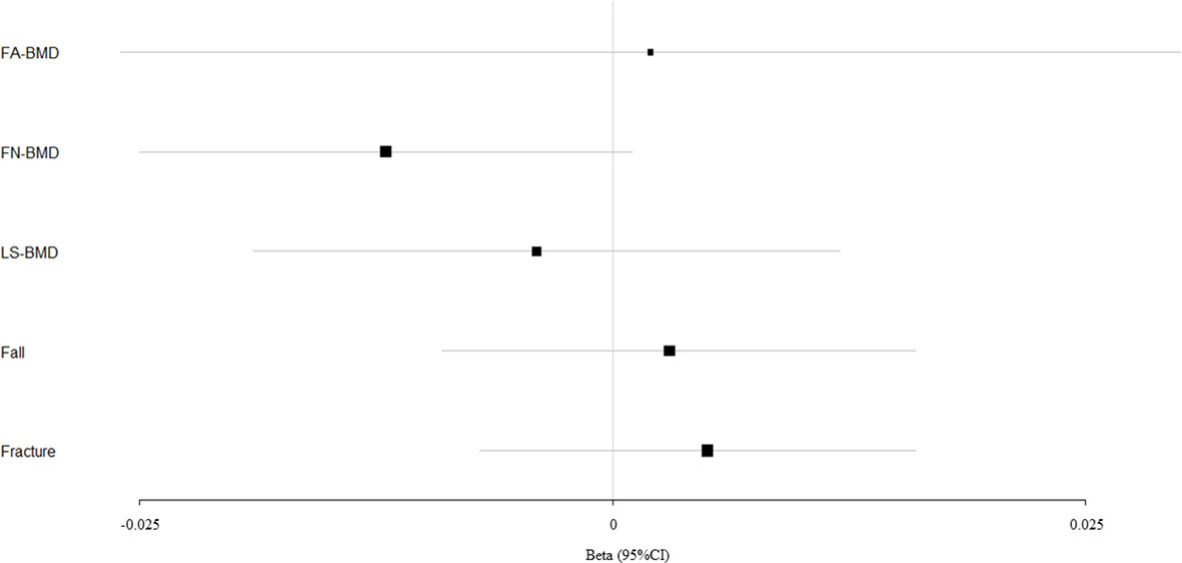
Mendelian randomization estimates for the associations between DBP and outcomes. FA-BMD, forearm BMD; FN-BMD, femoral neck BMD; LS-BMD, lumbar spine BMD; CI, confidence interval.

**Figure 3:**
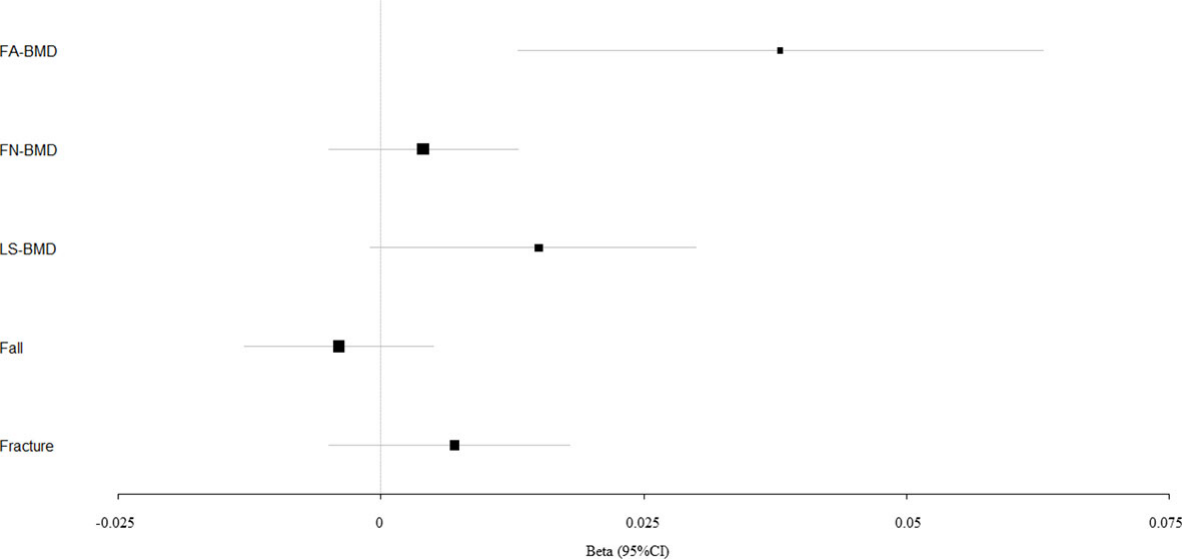
Mendelian randomization estimates for the associations between PP and outcomes. FA-BMD, forearm BMD; FN-BMD, femoral neck BMD; LS-BMD, lumbar spine BMD; CI, confidence interval.

We evaluated the causal effect of blood pressure including SBP (**Figure 1**), DBP (**Figure 2**), and PP (**Figure 3**) on FA-BMD, FN-BMD, and LS-BMD in the MR analysis (**Table 1**). High PP was significantly associated with improved FA-BMD (beta-estimate: 0.038, 95% confidence interval [CI]: 0.013 to 0.063, standard error [SE]: 0.013, P-value=0.003<Bonferroni correction P, **Figure 3**) in the IVW analysis, and this positive finding was supported by weighted-median analysis (beta-estimate: 0.034, 95% CI: 0.000 to 0.067, SE:0.017, P-value=0.046) and MR-Egger analysis (beta-estimate: 0.117, 95% CI: 0.026 to 0.208, SE:0.046, P-value=0.011). However, PP showed no remarkable influence on FN-BMD or LS-BMD based on the results of IVW, weighted-median, and MR-Egger analyses.

**Table 1:** Mendelian randomization estimates of blood pressure on outcomes.

Exposures	Outcomes	IVW							Weighted median				MR-Egger							
		Estimate	SE	95% CI	P-value	Q value	I^2^	Heterogeneity P value	Estimate	SE	95% CI	P-value	Estimate	SE	95% CI	P-value	Intercept	SE	95% CI	Pleiotropy P value
**Systolic blood pressure**	**FA-BMD**	-0.015	0.013	-0.041,0.011	0.258	27.962	28.50%	0.110	-0.017	0.017	-0.049,0.016	0.322	-0.011	0.063	-0.134,0.113	0.867	-0.002	0.022	-0.044,0.041	0.943
	**FN-BMD**	0.01	0.006	-0.001,0.021	0.064	16.592	0.00%	0.679	0.010	0.008	-0.005,0.025	0.200	0.012	0.025	-0.036,0.061	0.623	-0.001	0.009	-0.018,0.016	0.933
	**LS-BMD**	0.001	0.008	-0.014,0.016	0.921	28.841	30.70%	0.091	-0.003	0.010	-0.023,0.016	0.745	0.008	0.036	-0.062,0.078	0.819	-0.003	0.012	-0.027,0.022	0.832
	**Fall**	0.002 (OR 1.002)	0.004	-0.006,0.010 (OR 95% CI 0.994, 1.010)	0.620	28.455	33.20%	0.075	0.004	0.005	-0.006,0.014	0.472	0.023	0.018	-0.013,0.058	0.209	-0.007	0.006	-0.020,0.005	0.240
	**Fracture**	0.003 (OR 1.003)	0.005	-0.007,0.013 (OR 95% CI 0.993, 1.013)	0.583	20.620	7.90%	0.358	0.006	0.007	-0.007,0.020	0.331	-0.009	0.023	-0.054,0.037	0.711	0.004	0.008	-0.012,0.020	0.616
**Diastolic blood pressure**	**FA-BMD**	0.002	0.014	-0.026,0.030	0.876	33.732	0.00%	0.577	0.008	0.020	-0.031,0.046	0.702	0.015	0.059	-0.100,0.130	0.796	-0.003	0.012	-0.027,0.021	0.820
	**FN-BMD**	-0.012	0.007	-0.025,0.001	0.077	35.249	0.00%	0.504	-0.015	0.010	-0.034,0.004	0.127	0.048	0.028	-0.008,0.104	0.092	-0.013	0.006	-0.025,-0.001	0.030
	**LS-BMD**	-0.004	0.008	-0.019,0.012	0.666	37.504	4.00%	0.400	-0.008	0.011	-0.030,0.015	0.490	0.053	0.033	-0.011,0.118	0.106	-0.012	0.007	-0.026,0.001	0.076
	**Fall**	0.003 (OR 1.003)	0.006	-0.009,0.016 (OR 95% CI 0.991, 1.015)	0.577	82.365	57.50%	0.000	0.001	0.007	-0.011,0.014	0.821	0.009	0.027	-0.043,0.061	0.737	-0.001	0.006	-0.012,0.010	0.832
	**Fracture**	0.005 (OR 1.002)	0.006	-0.007,0.016 (OR 95% CI 0.993, 1.017)	0.434	33.437	0.00%	0.544	0.000	0.008	-0.016,0.016	0.991	-0.008	0.025	-0.057,0.041	0.753	0.003	0.005	-0.008,0.013	0.606
**Pulse pressure**	**FA-BMD**	0.038	0.013	0.013,0.063	0.003	41.576	15.80%	0.206	0.034	0.017	0.000,0.067	0.047	0.117	0.046	0.026,0.208	0.011	-0.022	0.012	-0.046,0.002	0.076
	**FN-BMD**	0.004	0.005	-0.005,0.013	0.335	26.248	0.00%	0.857	-0.006	0.007	-0.020,0.007	0.370	0.034	0.020	-0.005,0.072	0.086	-0.008	0.005	-0.017,0.002	0.125
	**LS-BMD**	0.015	0.008	-0.001,0.030	0.062	50.378	30.50%	0.045	0.014	0.010	-0.005,0.033	0.137	0.029	0.029	-0.029,0.087	0.325	-0.004	0.008	-0.019,0.011	0.617
	**Fall**	-0.004 (OR 0.996)	0.004	-0.013,0.005 (OR 95% CI 0.988, 1.004)	0.368	54.180	39.10%	0.012	-0.009	0.005	-0.019,0.001	0.095	-0.007	0.016	-0.038,0.024	0.660	0.001	0.004	-0.007,0.009	0.843
	**Fracture**	0.007 (OR 1.007)	0.006	-0.005,0.018 (OR 95% CI 0.995, 1.019)	0.246	45.821	28.00%	0.068	0.006	0.007	-0.008,0.020	0.408	0.022	0.021	-0.019,0.062	0.300	-0.004	0.006	-0.015,0.007	0.459

FA-BMD, forearm BMD; FN-BMD, femoral neck BMD; LS-BMD, lumbar spine BMD; IVW, inverse variance weighted; SE, standard error; CI, confidence interval; OR, odds ratio.

According to primary IVW analyses, SBP showed no MR association with FA-BMD (beta-estimate: −0.015, 95% CI: −0.041 to 0.011, SE:0.013, P-value=0.258), FN-BMD (beta-estimate: 0.010, 95% CI: −0.001 to 0.021, SE:0.006, P-value=0.064), or LS-BMD (beta-estimate: 0.001, 95% CI: −0.014 to 0.016, SE:0.008, P-value=0.921, **Figure 1**), while DBP also demonstrated no obvious impact on FA-BMD (beta-estimate: 0.002, 95% CI: −0.026 to 0.030, SE:0.014, P-value=0.876), FN-BMD (beta-estimate: −0.012, 95% CI: −0.025 to 0.001, SE:0.007, P-value=0.077), or LS-BMD (beta-estimate: −0.004, 95% CI: −0.019 to 0.012, SE:0.008, P-value=0.666, **Figure 2**) during the IVW analyses. These results were also confirmed by the weighted-median analysis and MR-Egger analysis.

### Causal Effect of Blood Pressure on Fall and Fracture

SBP, DBP, and PP showed null association with fall in the IVW (odds ratio [OR]: 1.002, 95% CI: 0.994 to 1.010; SE:0.004, P-value=0.620 for SBP, **Figure 1**; OR: 1.003, 95% CI: 0.991 to 1.015; SE:0.006, P-value=0.577 for DBP, **Figure 2**; OR: 0.996, 95% CI: 0.988 to 1.004; SE:0.004, P-value=0.368 for PP, **Figure 3**). Consistently, there was also no relationship between BP and fracture in the IVW analysis (OR: 1.003, 95% CI: 0.993 to 1.013; SE:0.005, P-value=0.258 for SBP, **Figure 1**; OR: 1.005, 95% CI: 0.993 to 1.017; SE:0.006, P-value=0.434 for DBP, **Figure 2**; OR: 1.007, 95% CI: 0.995 to 1.019; SE:0.006, P-value=0.246 for PP, **Figure 3**). These results were all confirmed by the weighted-median analysis and MR-Egger analysis.

### Evaluation of Assumptions and Sensitivity Analyses

Little evidence of directional pleiotropy was found for all models except for the association between DBP and FN-BMD (MR-Egger intercept P-value=0.03) (**Table 1**). The estimates from the weighted-median approach and MR-Egger analysis were all consistent with those of IVW models (**Table 1**).

Among the instrument variables, MR-PRESSO method only identified one outlier (rs72799341) for the association between DBP and fall, and one outlier (rs12628032) for the association between PP and fall. After excluding these outliers, DBP and PP still revealed no causal effect on the incidence of fall (OR: 1.002, 95% CI: 0.990 to 1.014, SE:0.006, P-value=0.801 for DBP; OR: 0.993, 95% CI: 0.985 to 1.001, SE:0.004, P-value=0.072 for PP, **Table 2**).

**Table 2:** Mendelian randomization estimates between blood pressure and outcomes after excluding outliers detected by MR-PRESSO.

Exposures	Outcomes	OR	SE	95% CI	P-value
**Diastolic blood pressure**	**Fall excluding one outlier (rs72799341)**	1.002	0.006	0.990,1.014	0.801
**Pulse pressure**	**Fall excluding one outlier (rs12628032)**	0.993	0.004	0.985,1.001	0.072

MR-PRESSO, Mendelian randomization pleiotropy residual sum and outlier test; SE, standard error; CI, confidence interval; OR, odds ratio.

## Discussion

In this two-sample MR analysis, we found the casual effect between high PP and improved FA-BMD (beta-estimate: 0.038, 95% CI: 0.013 to 0.063, SE:0.013, P-value=0.003<Bonferroni correction P), indicating that 1-SD increase in PP was associated with the improvement in FA-BMD levels by 0.038 g/cm^2^ (95% CI: 0.013 to 0.063). This positive finding was also confirmed by weighted median, MR-egger, and MR-PRESSO analyses. However, no causal association was seen between BP and other outcomes (i.e., FN-BMD, LS-BMD, fall, and fracture).

Hypertension has important association with alterations in calcium metabolism, including increased calcium loss, compensatory activation of parathyroid gland, and increased movement of calcium from the bones (32). Long-lasting impairment effect of hypertension on calcium homeostasis may result in age-related excessive reduction of BMD and fracture (32). Previous studies explored the association between BP and BMD, but reported conflicting results (14–16).

In one cross-sectional study involving 270 postmenopausal Turkish women, hypertension was found to be significant predictors of osteopenia in a multivariate analysis (OR: 2.541, 95% CI: 1.46–3.48, P=0.003) (14). A retrospective analysis of 586 postmenopausal women with a mean age of 60.8 ± 8.8 years old revealed that hypertension was associated with low spine BMD in postmenopausal women (16). In contrast, another cross-sectional study was conducted among 4,058 premenopausal and postmenopausal women aged 40 years or older (number=991 and 3,067, respectively), and the results revealed no link between BP (i.e., SBP and DBP) and BMD (15). These studies did not involve male patients and were indeed contradictory. These inconsistent results may result from small patient sample, confounding factors, and the limitation of study design.

Our two-sample MR analysis involved 53,236 European individuals for the association with BMD, 451,179 European individuals for fall susceptibility, and up to 1.2 million individuals from European descent for fracture. The results revealed the important causal effect of high PP on improved FA-BMD (P=0.003), which was confirmed by multiple sensitivity analyses. The association between PP and BMD was explored after adjusting for sex, age, age^2^, and weight (22). In addition, we found no obvious MR association between BP and other outcomes (i.e., FN-BMD, LS-BMD, fall, and fracture).

It is very interesting to confirm that genetically high PP shows strong MR association with improved BMD. PP is defined as SBP minus DBP. High PP largely results from large-artery stiffness, while decreased PP is caused by low stroke volume, such as congestive heart failure and aortic valve stenosis (33). Vascular smooth muscle cells (VSMC) have important roles in regulating arterial stiffness by overproducing various extracellular matrix components (e.g., collagen and elastin), which provide biomechanical, structural integrity, and signaling regulation of the extracellular matrix components to maintain vascular homeostasis (34, 35). However, patients with high PP have vascular calcification and increased arterial stiffness, which increase the expression of bone markers such as alkaline phosphatase (ALP) and type 1 collagen. These factors also improve the osteogenic differentiation and mineralization for bone formation and increased BMD (36).

Forearm BMD at the 1/3 radial site is commonly used to improve the prediction of hip fractures when considered together with FN-BMD. Forearm BMD represents the BMD of combined trabecular and cortical bone structures, indicating the better improvement of BMD than other sites of BMD after effective anti-osteoporosis treatments, which may account for the positive MR association only between PP and forearm BMD (37). In addition, 1,032 men and 1,701 women aged 50 years and older were included in the Dubbo Osteoporosis Epidemiology Study, and hypertension may be an independent risk factor for fragility fracture (hazard ratio, 1.49; 95% CI, 1.13–1.96) after adjusting for BMD and covariates (38). However, our two-sample MR analysis confirmed null association between BP and fracture.

To the best of our knowledge, this is the first two-sample MR study to find the positively casual association between PP and FA-BMD. The summary statistics of outcome phenotypes are retrieved from GWAS or genome-wide meta-analysis with huge sample size. This two-sample MR method allows for the estimation of the causal effects of BP on all outcomes while at the same time minimizing reverse causation bias and confounding factors. The intercepts for the MR-Egger analysis, except for the association between DBP and FN-BMD, indicate that there is no directional pleiotropy to influence other causal associations.

Several limitations should be taken into consideration. Firstly, there is some heterogeneity between DBP (or PP) and fall, which may be caused by the selection of some instrumental variables. Secondly, this MR study reveals the potential causal effect of PP on FA-BMD, but null association is observed between BP and FN-BMD (or LS-BMD). The factors of this inconsistency remain elusive. Thirdly, it is not feasible to perform the MR analysis based on different age stratums because of the limitation of GWAS summary statistics. Fourthly, the MR analysis is restricted in the European-ancestry population, which may limit the generalizability of our finding to other populations.

## Conclusion

This two-sample MR reveals the potential causal effect of high PP on improved FA-BMD, suggesting the protective role of high PP for osteoporosis.

## Data Availability Statement

The original data were available in GEnetic Factors for OSteoporosis Consortium (http://www.gefos.org/) and the UK Biobank (https://www.ukbiobank.ac.uk/). The instructions to download data from (http://www.gefos.org/) are presented in **Datasheet 3**.

## Author Contributions

BH, ZQ, and QL conducted study design. BH, LFY and MZZ conducted data collection and statistical analysis. BH, ZQ, QL, and YO conducted data interpretation, manuscript preparation, and literature search. BH and QL conducted funds collection. All authors contributed to the article and approved the submitted version.

## Funding

This study was funded by Natural Science Foundation of Chongqing (cstc2019jcyj-msxmX0836), National Natural Science Foundation of China (81701382), Foundation of The First Affiliated Hospital of Chongqing Medical University (PYJJ2018-13), and Chongqing Yuzhong Nature Science Foundation of China (Grant No. 2018114).

## Conflict of Interest

The authors declare that the research was conducted in the absence of any commercial or financial relationships that could be construed as a potential conflict of interest.

## Publisher’s Note

All claims expressed in this article are solely those of the authors and do not necessarily represent those of their affiliated organizations, or those of the publisher, the editors and the reviewers. Any product that may be evaluated in this article, or claim that may be made by its manufacturer, is not guaranteed or endorsed by the publisher.
