# Supplementary material for: Causal Effect of Blood Pressure on Bone Mineral Density and Fracture: A Mendelian Randomization Study
Source: Front Endocrinol (Lausanne). 2021 Aug 4;12:716681. doi: 10.3389/fendo.2021.716681 (PMC8371435; doi:10.3389/fendo.2021.716681)
Supplement: Supplementary file 3 [file DataSheet_3.pdf]

Detail procedures to download the GWAS summary data of all outcomes:
1 Click "http://www.gefos.org/"
2 Then, click "Resources"---"Gefos Data Release"

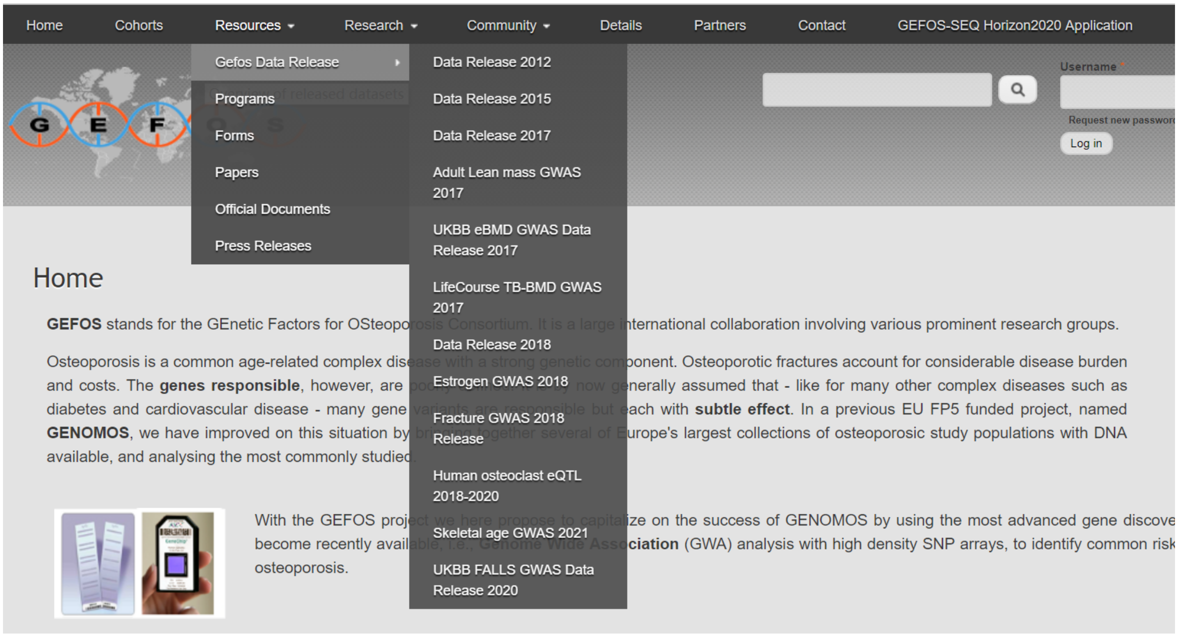

3 For GWAS summary data of forearm BMD, femoral neck BMD and lumbar spine BMD, please click "Data release 2015".

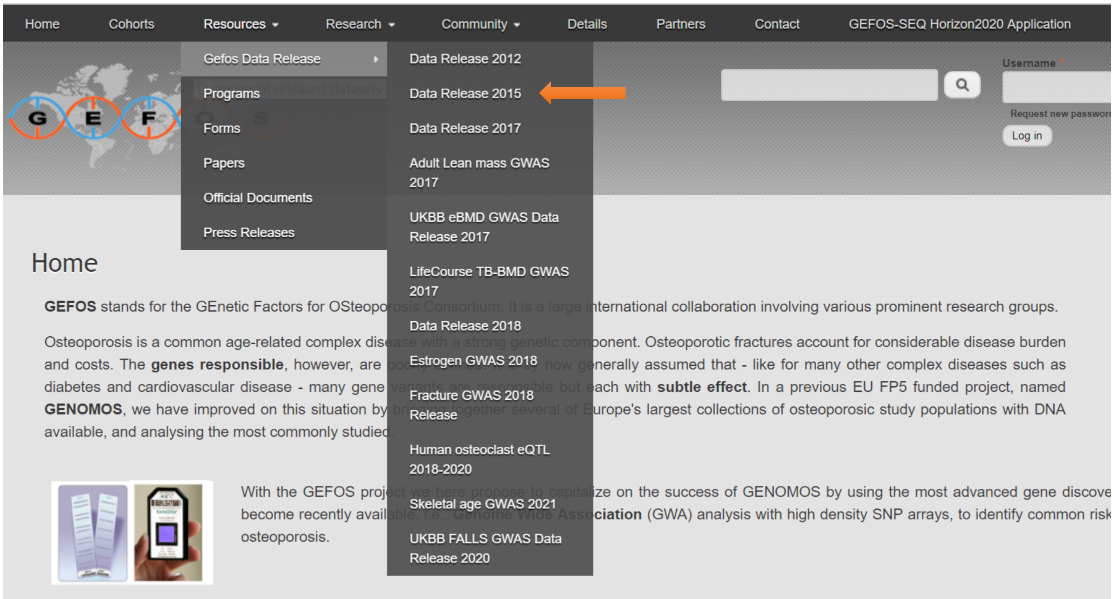

4 Then, click "fa2stu.MAF0_.005.pos_.out_.gz",
"fn2stu.MAF0_.005.pos_.out_.gz",
"ls2stu.MAF0_.005.pos_.out_.gz"
at the bottom of webpage to down the GWAS summary data of forearm BMD, femoral neck BMD and lumbar spine BMD, separately.

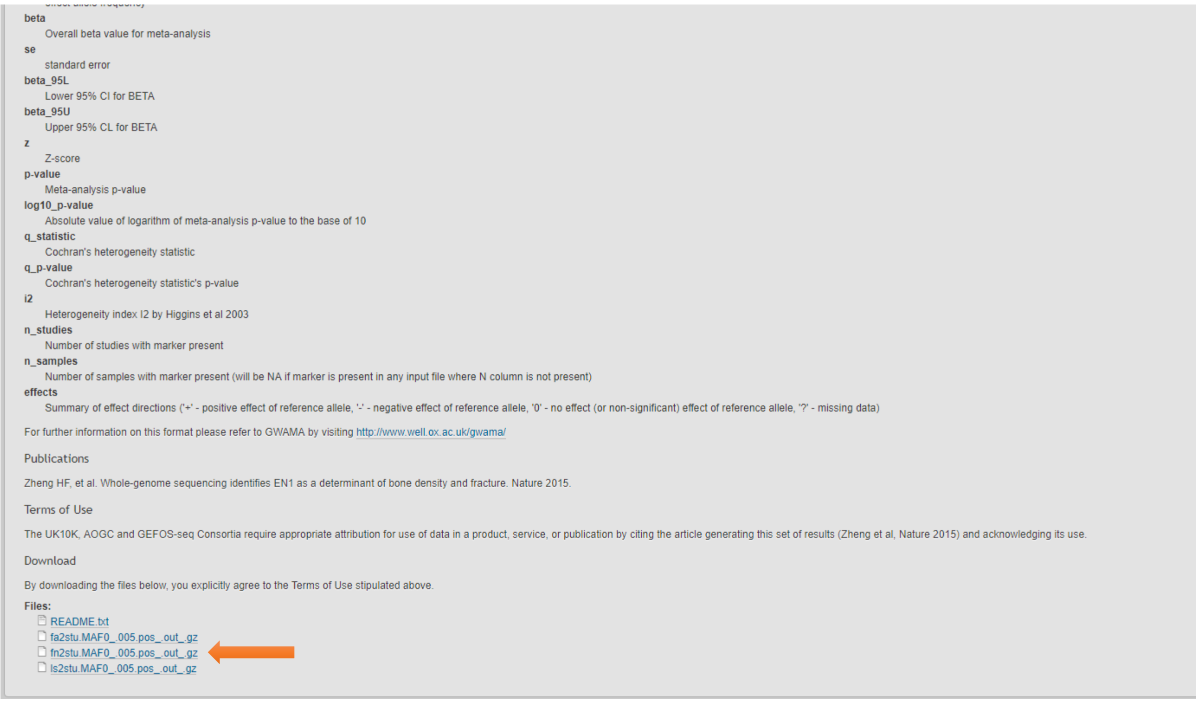

5 For GWAS summary data of fall, please click “UKBB FALLS GWAS Data Release 2020”.

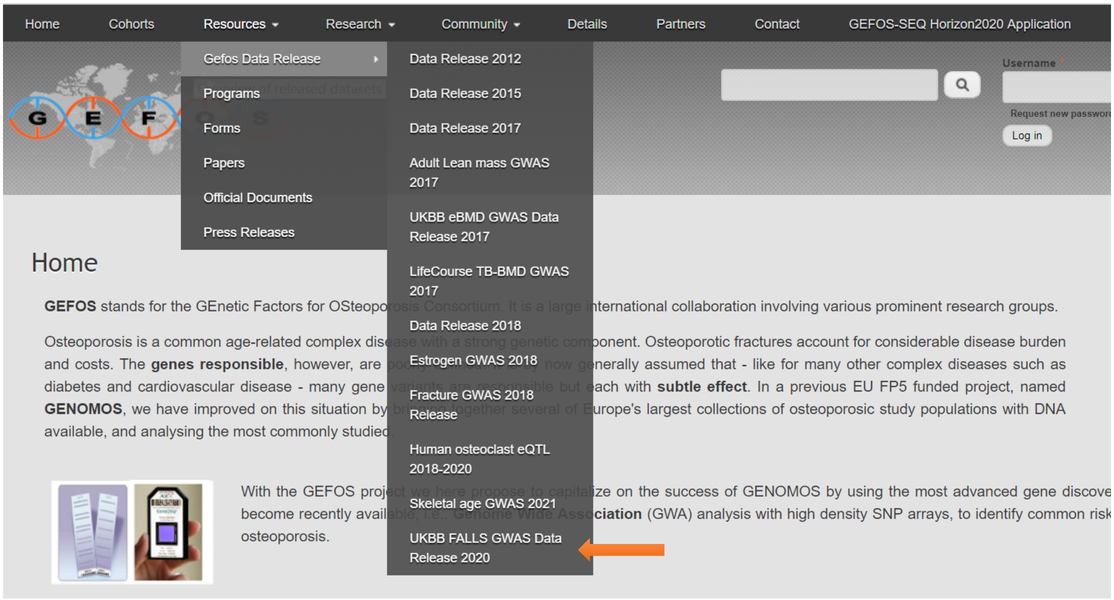

6 Then, click “UKBB_falls_gwas_MAF001_INF003.txt.gz” at the bottom of webpage to download the GWAS summary data of fall.

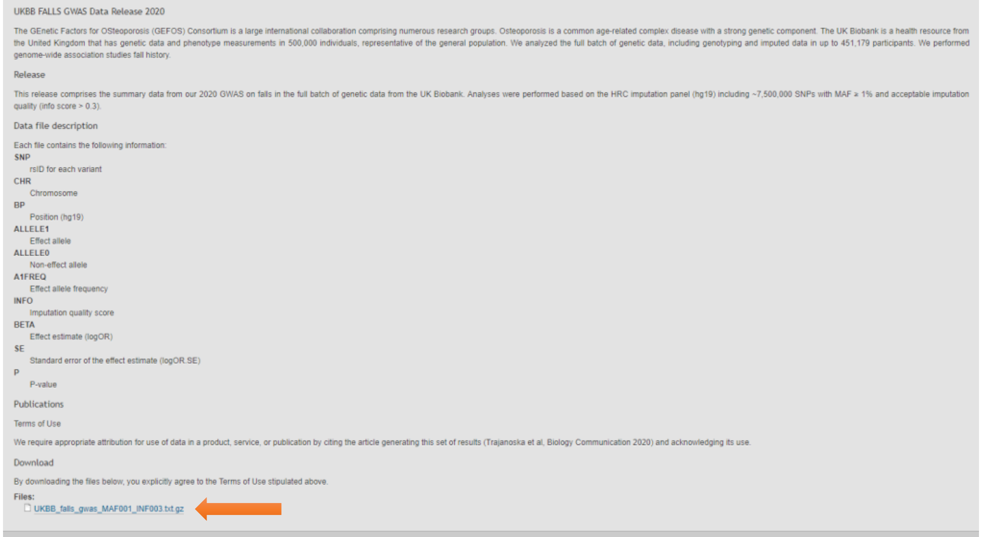
UKBB FALLS GWAS Data Release 2020

The GEnetic Factors for OSteoporosis (GEFOS) Consortium is a large international collaboration comprising numerous research groups. Osteoporosis is a common age-related complex disease with a strong genetic component. The UK Biobank is a health resource from the United Kingdom that has genetic data and phenotype measurements in 500,000 individuals, representative of the general population. We analyzed the full batch of genetic data, including genotyping and imputed data in up to 451,179 participants. We performed genome-wide association studies fall history.

Release

This release comprises the summary data from our 2020 GWAS on falls in the full batch of genetic data from the UK Biobank. Analyses were performed based on the HRC imputation panel (hg19) including ~7,500,000 SNPs with MAF ≥ 1% and acceptable imputation quality (info score > 0.3).

Data file description

Each file contains the following information:

SNP
rsID for each variant
CHR
Chromosome
BP
Position (hg19)
ALLELE1
Effect allele
ALLELE0
Non-effect allele
A1FREQ
Effect allele frequency
INFO
Imputation quality score
BETA
Effect estimate (logOR)
SE
Standard error of the effect estimate (logOR.SE)
P
P-value

Publications

Terms of Use

We require appropriate attribution for use of data in a product, service, or publication by citing the article generating this set of results (Trajanoska et al, Biology Communication 2020) and acknowledging its use.

Download

By downloading the files below, you explicitly agree to the Terms of Use stipulated above.

Files:
UKBB_falls_gwas_MAF001_INF003.txt.gz

7 For GWAS summary data of fracture, please click "Data Release 2018".

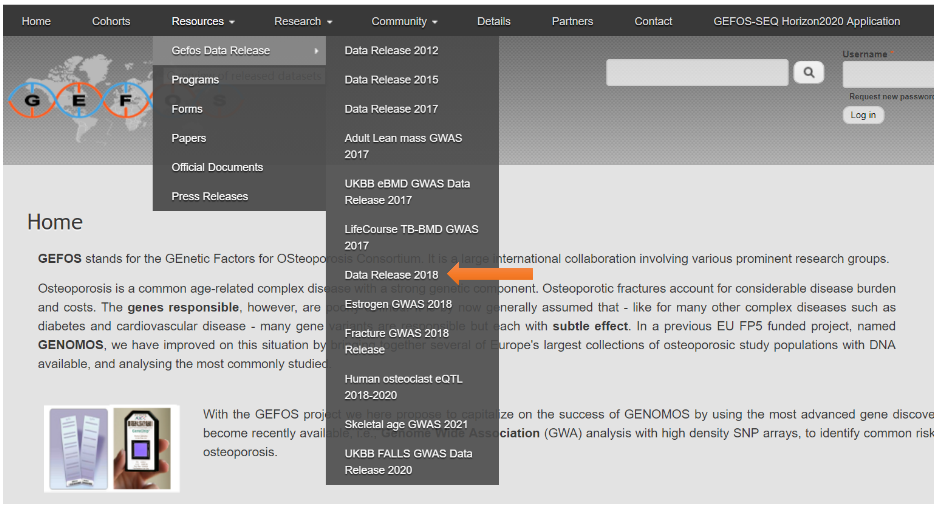

8 Then, click "Morrisetal2018.NatGen.SumStats.tar.gz" at the bottom of webpage to download GWAS summary data of fracture.

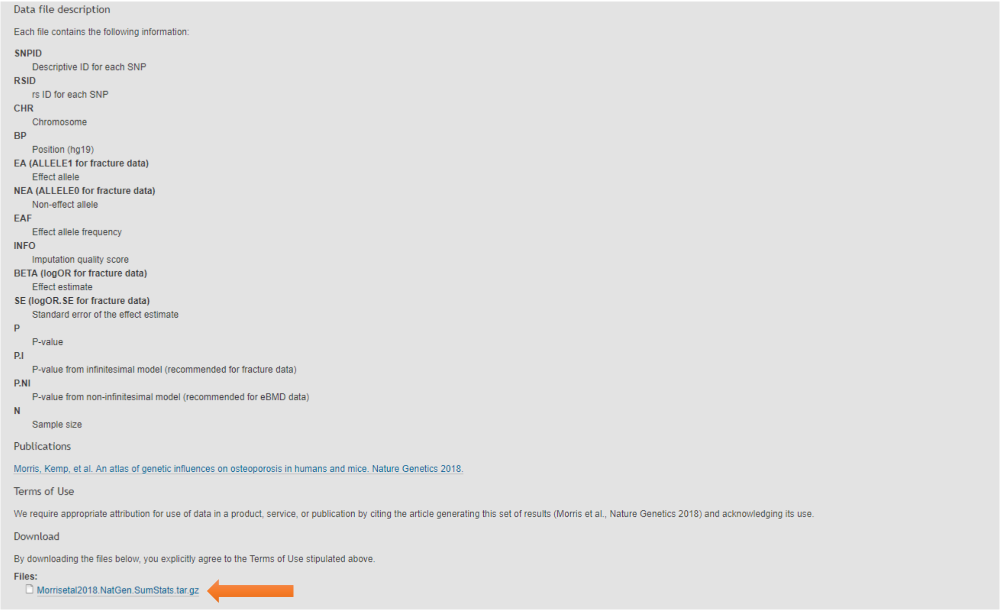
Data file description

Each file contains the following information:

SNPID
Descriptive ID for each SNP
RSID
rs ID for each SNP
CHR
Chromosome
BP
Position (hg19)
EA (ALLELE1 for fracture data)
Effect allele
NEA (ALLELE0 for fracture data)
Non-effect allele
EAF
Effect allele frequency
INFO
Imputation quality score
BETA (logOR for fracture data)
Effect estimate
SE (logOR.SE for fracture data)
Standard error of the effect estimate
P
P-value
P.I
P-value from infinitesimal model (recommended for fracture data)
P.NI
P-value from non-infinitesimal model (recommended for eBMD data)
N
Sample size

Publications

Morris, Kemp, et al. An atlas of genetic influences on osteoporosis in humans and mice. Nature Genetics 2018.

Terms of Use

We require appropriate attribution for use of data in a product, service, or publication by citing the article generating this set of results (Morris et al., Nature Genetics 2018) and acknowledging its use.

Download

By downloading the files below, you explicitly agree to the Terms of Use stipulated above.

Files:
Morrisetal2018.NatGen.SumStats.tar.gz
